# Reliability of the Fluoroscopic Assessment of Load-Induced Glenohumeral Translation during a 30° Shoulder Abduction Test

**DOI:** 10.3390/biomechanics2020020

**Published:** 2022-05-19

**Authors:** Eleonora Croci, Marina Künzler, Sean Börlin, Franziska Eckers, Corina Nüesch, Daniel Baumgartner, Andreas Marc Müller, Annegret Mündermann

**Affiliations:** 1 Department of Biomedical Engineering, University of Basel, 4001 Basel, Switzerland; 2 Department of Orthopaedics and Traumatology, University Hospital Basel, 4031 Basel, Switzerland; 3 Department of Clinical Research, University of Basel, 4031 Basel, Switzerland; 4 Department of Spine Surgery, University Hospital Basel, 4031 Basel, Switzerland; 5 IMES Institute of Mechanical Systems, Zurich University of Applied Sciences ZHAW, 8400 Winterthur, Switzerland

**Keywords:** shoulder, rotator cuff, glenohumeral instability, humeral head migration, fluoroscopy, abduction

## Abstract

Rotator cuff tears are often linked to superior translational instability, but a thorough understanding of glenohumeral motion is lacking. This study aimed to assess the reliability of fluoroscopically measured glenohumeral translation during a shoulder abduction test. Ten patients with rotator cuff tears participated in this study. Fluoroscopic images were acquired during 30° abduction and adduction in the scapular plane with and without handheld weights of 2 kg and 4 kg. Images were labelled by two raters, and inferior-superior glenohumeral translation was calculated. During abduction, glenohumeral translation (mean (standard deviation)) ranged from 3.3 (2.2) mm for 0 kg to 4.1 (1.8) mm for 4 kg, and from 2.3 (1.5) mm for 0 kg to 3.8 (2.2) mm for 4 kg for the asymptomatic and symptomatic sides, respectively. For the translation range, moderate to good interrater (intra-class correlation coefficient ICC [95% confidence interval (CI)]; abduction: 0.803 [0.691; 0.877]; adduction: 0.705 [0.551; 0.813]) and intrarater reliabilities (ICC [95% CI]; abduction: 0.817 [0.712; 0.887]; adduction: 0.688 [0.529; 0.801]) were found. Differences in the translation range between the repeated measurements were not statistically significant (mean difference, interrater: abduction, −0.1 mm, *p* = 0.686; adduction, −0.1 mm, *p* = 0.466; intrarater: abduction 0.0 mm, *p* = 0.888; adduction, 0.2 mm, *p* = 0.275). This method is suitable for measuring inferior-superior glenohumeral translation in the scapular plane.

## 1 Introduction

Although rotator cuff tears are clinically linked to abnormal shoulder joint kinematics [1–4], a thorough understanding of glenohumeral motion is still lacking, particularly during the initial phase of arm abduction. Reported changes in shoulder translation in patients with rotator cuff tears are inconclusive [1–5]. Moreover, only few studies have analysed shoulder kinematics under loaded conditions that are comparable to daily activities [3,6–9], and the effect of a load on shoulder kinematics in patients with rotator cuff tears has not been investigated. For instance, shoulder kinematics in healthy subjects during arm abduction with a 1 kg weight was studied by Chen et al. [6], who found no changes in the humeral head position from 0° to 135° abduction. In contrast, Teyhen et al. [9] found a more superior humeral head position during abduction, and similar results were reported by Chopp et al. [7] up to 90° abduction. Only Nishinaka et al. [8] compared shoulder kinematics of loaded (3 kg) and unloaded arm abduction, but did not assess the differences between conditions, and reported an average of 2 mm translation from 0° to 90° abduction.

The clinical manifestation of rotator cuff tears varies largely among patients: some patients have no complaints and the diagnosis is an incidental finding, while other patients have severe pain and limited active range of motion that can be marginally restored with conservative treatment, such as physiotherapy, cortisone injections, or non-steroidal anti-inflammatory drugs [10]. One possible reason for the variability in symptoms and functional limitations could be the superior translational instability due to an insufficient joint centring as the consequence of a dysfunctional rotator cuff. This would occur especially in complete supraspinatus tendon tears, but also in partial tears where the supraspinatus' line of action could be compromised, leading to joint instability. This superior translation may cause pain due to increased pressure on the periphery of the glenoid cavity and the labrum, and possibly the impingement of the long biceps tendon or the supraspinatus tendon [11]. Hence, shoulder motion might be restricted because of the subsequent interference of the humerus with the acromion.

Previous studies [3,4,6,7,9,12–14] have used static measurement techniques (conventional radiographs) to describe shoulder kinematics. However, the findings of static measurements might not be generalizable to dynamic conditions as isometric and concentric muscle contractions could influence shoulder kinematics differently. More recently, shoulder kinematics have been reported during scapular plane abduction using 3D-to-2D model-to-image registration techniques, where computer tomography (CT)-derived bone models of the humerus and scapula are matched to the profile of the bones in the fluoroscopic images [15–21]. Although this method can provide accurate 3D measurements, CT scans are required and, hence, patients are exposed to additional ionizing radiation. Because CT scans of the shoulder have, on average, an effective dose of 10.83 mSv, corresponding to a lifetime cancer risk of 0.60–0.73 [22], avoiding this examination would be beneficial for patients.

Because of the increased interest in the inferior–superior glenohumeral translation in patients with rotator cuff tears and the importance of the applicability in the clinic, single-plane fluoroscopy with a low radiation dose (below 0.01 mSv) may be employed for assessing shoulder kinematics during scapular plane abduction with expected good reliability. The aim of this study was to assess the reliability of fluoroscopically measured glenohumeral translation during an unloaded and loaded 30° abduction test in the scapular plane in patients with rotator cuff tears.

## 2 Materials and Methods

### 2.1 Participants

Ten patients with symptomatic rotator cuff tears (6 men and 4 women; mean (standard deviation) age: 65.6 (10.3) years; height: 175 (8) cm; body mass: 79.5 (15.8) kg; body mass index (BMI): 26.2 (5.5) kg/m^2^) participated in this study. Five patients were diagnosed with a complete tear of the supraspinatus tendon (N = 3 combined with other rotator cuff tears) and five patients with a partial tear of the supraspinatus tendon (N = 3 combined with other rotator cuff tears). The contralateral side of all patients was asymptomatic with no known previous injuries and no limitation to the range of motion. The symptomatic side was the dominant side in eight of ten patients and, compared to the contralateral side, all had signs of a deficient range of motion. All patients had already undergone physiotherapy sessions; seven patients reported mild pain (visual analogue scale <5) and three patients had no pain in the shoulder. Patients were recruited from the Orthopaedics and Traumatology Clinic carrying out this study. Patients between 45 and 85 years old were included if they had a unilateral rotator cuff tear of at least the supraspinatus tendon (either a partial or complete tear). Exclusion criteria were: BMI > 35 kg/m^2^; inability to provide informed consent; prior operative treatment of the ipsilateral upper extremity; clinical history of the contralateral glenohumeral joint (e.g., injuries or persistent pain); neuromuscular disorders affecting upper limb movement; and other pathologies influencing shoulder joint mobiliy. Prior to data collection, informed consent was obtained. This study was approved by the regional ethics board (Ethikkomission Nordwestschweiz EKNZ 2021-00182) and conducted in accordance with the Declaration of Helsinki (2013).

### 2.2 Image Acquisition

**Figure 1:**
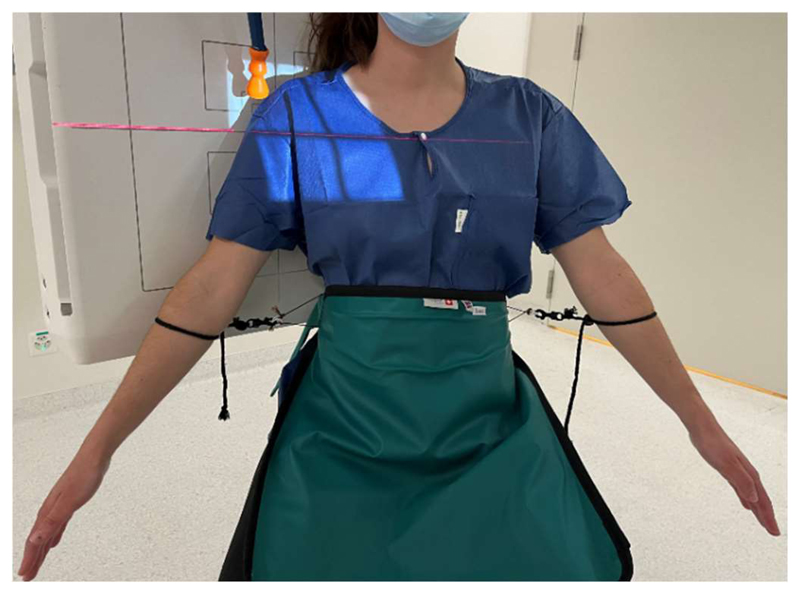
Patient with the arm at maximal amplitude. The patient is positioned against the fluoroscopic device for the acquisition of images in the scapular plane. The string attached to the lower arm controls the maximal arm amplitude during the abduction test.

Single-plane fluoroscopic images (Multitom Rax, Siemens Healthineers, Erlangen, Germany) were acquired during a 30° shoulder abduction test in the scapular plane. Subjects were seated in upright posture on a stool without a backrest. They were instructed to keep the elbows extended and hands in a neutral position during arm abduction. Subjects were asked to abduct their arms to 30° in the scapular plane and then to return to the initial position. Preceding data acquisition, subjects were asked to perform practice movements (without handheld weights) in the scapular plane until the correct movement was achieved. After a rest period of at least 30 s, data for three conditions were collected: without additional weight, and in a randomized order with handheld weights of 2 kg and 4 kg, to resemble common situations of daily, occupational, or recreational activities. Rest time between each condition was at least 30 s. Images were acquired first for the right shoulder independent of the symptomatic side. After a rest period of at least 1 min, the same tests were repeated in the same order and images were acquired for the left shoulder. To control the maximal amplitude of arm abduction, a string (anchored to the protective shield) was attached to the lower arm and adjusted to the desired maximum position using a goniometer (Figure 1). Orientation in the scapular plane was checked initially by fluoroscopy. Images were captured with a pulse rate of 3 Hz to minimize radiation exposure. Verbal commands were given to the subjects to ensure a comparable movement speed. On average, each 30° abduction and adduction cycle lasted 9.2 (1.3) s. A reference ball (Ø = 25 mm) was placed in the field of view and used to calibrate the image dimensions.

### 2.3 Assessment of Glenohumeral Translation

**Figure 2:**
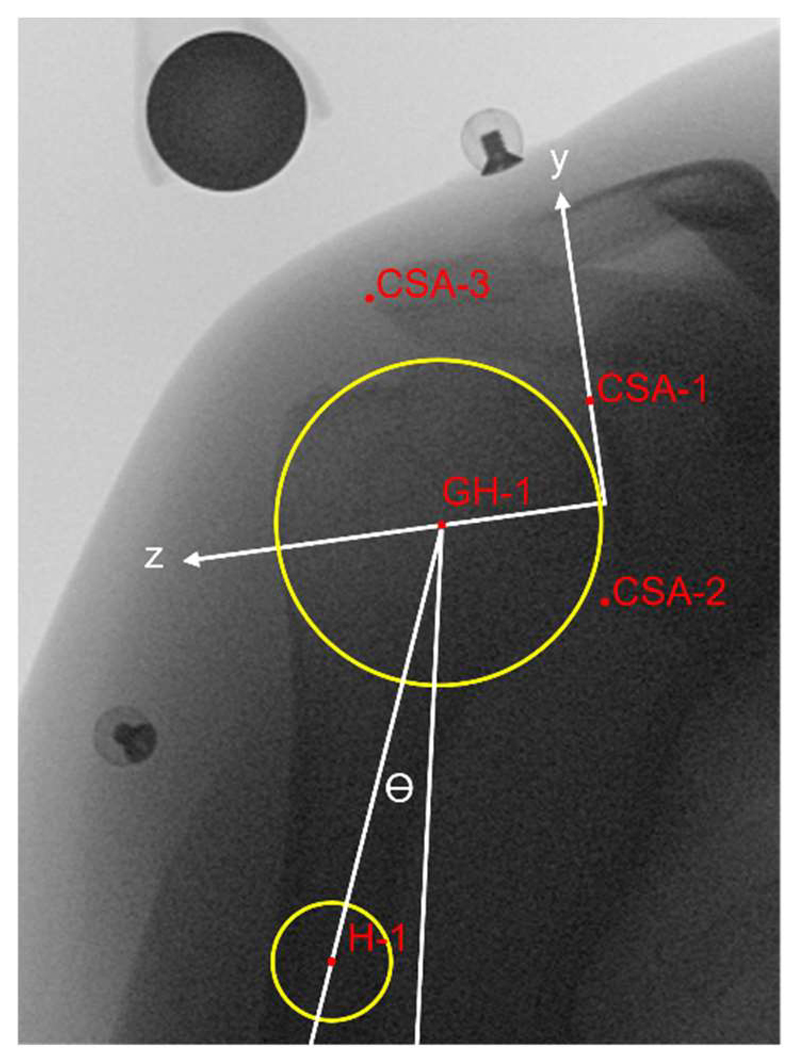
Example of a labelled image at the initial position with the glenoid coordinate system. The glenohumeral head joint centre (GH-1) is defined as the centre of a best-fit circle on the humeral head. The axis GH-1-H-1 Is used to measure the arm abduction angle (θ). H1 is the centre of a circle fitted to the humeral shaft (set as distally as possible). The critical shoulder angle (CSA) points are used as a reference for the translation measurements [23].

Each fluoroscopy image of these abduction tests was manually labelled (3D Slicer, https://www.slicer.org/ (accessed on 9 August 2021), Fedorov et al., 2012) by two raters (rater 1 and rater 2) after an initial training period. All the images were also labelled twice by rater 2 (two repetitions) after all the patients' images had been labelled once. Raters were blinded to the diagnosis and labelled patient image sets in random order. Reference markers for the glenohumeral joint centre and the critical shoulder angle [23], as shown in Figure 2, were placed on the first image of each sequence and then tracked and adjusted on the subsequent images (keeping humeral head radius constant).

The glenohumeral joint centre was determined as the geometric centre of a circle comprising the articulating surface of the humeral head (similar to the sphere method in the case of 3D images) [4,24–27]. All data were processed with MATLAB 2021b (The Math-works Natick, MA, USA). A 3-span moving average filter was applied on the raw data. Glenohumeral translation during arm abduction was measured as the inferior–superior component of a glenoid coordinate system [9], with orientation defined at the initial position (Figure 2). No translation was assumed at the initial position for all conditions. Inferior and superior glenohumeral translations were represented by negative and positive values, respectively. The abduction angle was measured as the angle between a line passing through the glenohumeral joint centre and the humerus shaft midpoint (Figure 2) and the vertical. The maximum abduction angle (maxAA) was calculated, and the translation range during abduction (from the initial position to maxAA) and during adduction (from maxAA to the end position) movements was computed. Thus, a positive translation during abduction would mean that the humeral head had moved upwards and a negative translation during adduction would indicate that the humerus had returned to the initial position.

### 2.4 Statistical Analysis

All statistical analyses were performed in R statistical software (R Core Team, 2019). All data were tested for normality with the Shaptro–Wilk's test, and since they were normally distributed, parametric statistical tests were applied. Limits of agreements for the range of glenohumeral translation during abduction and adduction between raters (1 vs. 2 and 2 vs. 2 rep.) were analysed and are presented as Bland–Altman plots [28]. Paired *t*-tests were used to detect the differences in the translation ranges between and within the raters (1 vs. 2 and 2 vs. 2 rep.), and between sides and across the load (mean values of raters 1 and 2). Intra-class correlations (two-way model, absolute agreement, single measurement; ICC(A,1)) for the interrater reliability and intrarater reliability and their 95% confidence interval (CI) were computed. Reliability was considered moderate for 0.5 ≤ ICC < 0.75 and good for 0.75 ≤ ICC < 0.9. Lower and greater ICC values would therefore be indicative of poor (ICC < 0.5) and excellent (ICC ≥ 0.9) reliability [29]. The significance level was set a priori to *p* < 0.05.

## 3 Results

**Figure 3:**
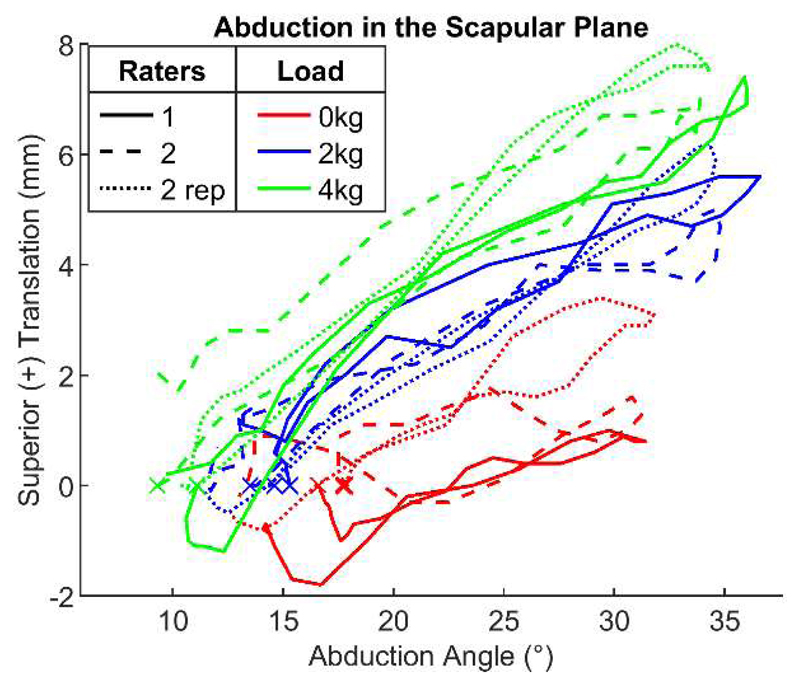
Example of tracked glenohumeral translations during arm abduction for all raters. ‘x’ indicates the initial position of the movement. Positive translation values represent a superior translation of the glenohumeral joint centre.

In Table 1, mean values (average between raters 1 and 2) are reported for the translation range during abduction and adduction movements for all patients for the asymptomatic and the symptomatic side. During abduction, glenohumeral translation ranged from 3.3 (2.2) mm for 0 kg to 4.1 (1.8) mm for 4 kg, and from 2.3 (1.5) mm for 0 kg to 3.8 (2.2) mm for 4 kg, for the asymptomatic and symptomatic sides, respectively (see Table 1). During adduction, glenohumeral translation ranged from −3.4 (1.9) mm for 0 kg to −3.9 (1.3) mm for 4 kg, and from −1.9 (1.2) mm for 0 kg to −3.4 (2.3) mm for 4 kg, for the asymptomatic and symptomatic sides, respectively. None of the differences between sides and loads were statistically significant during abduction (*p* > 0.05). During adduction, differences between 0 kg and 4 kg of the symptomatic side and between sides for 0 kg were significant (*p* = 0.030 and *p* = 0.019, respectively). For this repeatability study, the distinction between symptomatic/asymptomatic side and different handheld weights was not further considered. Thus, all data points of the translation range during abduction and adduction were considered together. Figure 3 shows the glenohumeral translation of one shoulder during the unloaded (0 kg) and loaded (2 kg and 4 kg) abduction tests in the scapular plane, as assessed by all raters (1, 2, and 2 rep.). Overall, the measured translations by the different raters were comparable, and the effect of the handheld weight was visible during arm abduction.

### 3.1 Interrater Reliability

**Figure 4:**
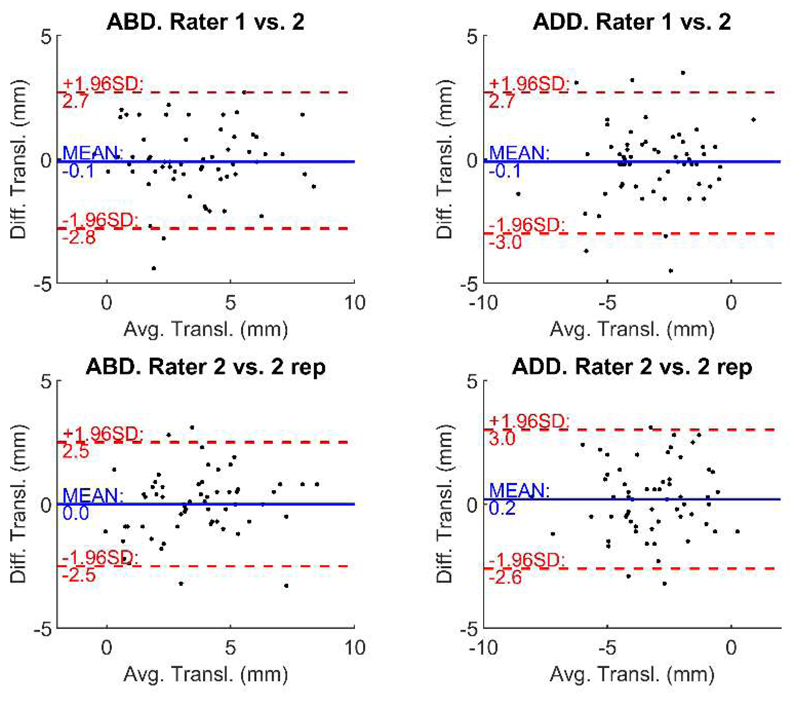
Bland-Altman plots showing the limits of agreements of the translation range for abduction and adduction for the interrater and intrarater reliability. ABD.—Abduction; ADD.—Adduction; Diff.—Difference; Avg.—Average; Transl.—Translation.

The translation range did not differ between the repeated measurements of raters 1 and 2 during abduction (*p* = 0.686) or adduction (*p* = 0.466). The corresponding limits of agreements are shown in Figure 4. Good reliability was found for the translation range during abduction (ICC(A,1) = 0.803, 95% confidence interval (CI): [0.691; 0.877]), while a moderate reliability during adduction was observed (ICC(A,1) = 0.705, 95% CI: [0.551; 0.813]).

### 3.2 Intrarater Reliability

The translation range did not differ between repeated measurements of the same rater (2 vs. 2 rep.) during abduction (*p* = 0.888) and adduction (*p* = 0.275). Good reliability was found for the translation range during abduction (ICC(A,1) = 0.817, 95% CI: [0.712; 0.887]), whereas moderate reliability during adduction was observed (ICC(A,1) = 0.688, 95% CI: [0.529; 0.801]).

## 4 Discussion

In this study, a simple method for measuring inferior–superior glenohumeral translation during a 30° abduction test in the scapular plane is presented, which—in contrast to 3D-to-2D model-to-image registration techniques—does not require additional CT-derived bone models [16–18,20,21]. The method comprises identifying some anatomical points of the glenohumeral centre (as the geometric centre of a 'best-fit' circle) and the inferior and superior edges of the glenoid [23] on the fluoroscopic images taken during scapular plane abduction. The method used is similar to the methods previously reported for static measurements [3,4,6,7,9,12]. However, we considered the entire motion of the abduction tests.

Glenohumeral translations during 30° abduction tests in the scapular plane without additional weight and with handheld weights of 2 kg and 4 kg were measured for the symptomatic and asymptomatic sides of ten patients with complete or partial tears of the supraspinatus tendon, and interrater and intrarater reliabilities of the translation ranges during abduction and adduction movements were investigated. For the unloaded abduction test, superior glenohumeral translations of 3.3 mm and 2.3 mm were found for the asymptomatic and symptomatic sides, respectively. These values are in line with the studies of Giphart et al. [16] and Kozono et al. [18]. Giphart et al. reported a 3.0 mm superior translation at 30° arm abduction in a young healthy group using a dynamic biplane fluoroscopy system [16]. In comparison, Kozono et al. found a superior glenohumeral translation of about 2 mm at 30° of scapular plane abduction in elderly patients with rotator cuff tears using 3D-to-2D model-to-image registration techniques [18]. Hence, the presented method is reproducible for translation both during abduction and during adduction. Indeed, the ICC measurements indicated, overall, a moderate to good reliability for the computed variables between and within raters. Moreover, there were no statistically significant differences between repeated measurements for the translation ranges, neither between nor within raters. Based on these results, assessing the reliability of the measured glenohumeral translations by two raters labelling the fluoroscopic images can be considered sufficient. In the presented setup, the limits of agreement for the glenohumeral translations were within 3 mm for both interrater and intrarater reliability during the abduction and adduction movements. Hence, accounting for measurement errors, differences in glenohumeral translation during abduction and adduction within a person (e.g., between sides, between conditions, or before or after treatment) or between persons (e.g., patients versus controls) exceeding 3 mm should be considered relevant.

In this study, radiation exposure was kept at a minimum (overall effective dose below 0.01 mSv) and, therefore, a pulse rate of 3 Hz was used for image acquisition. This low pulse rate led to some images being blurred because of arm motion, which may have influenced the raters' decision on where to place markers. Increasing the pulse rate will increase image resolution, presumably resulting in even higher reliability. However, one must consider the trade-off between improving image quality and increasing radiation exposure for patients when choosing the pulse rate. With better image quality, it might also be possible to use an automatic detection algorithm potentially further increasing the repeatability of the marker placement.

Only ten patients with rotator cuff tears were examined, making assumptions about differences in glenohumeral translation between the symptomatic and asymptomatic sides difficult; however, this was not the scope of this study. Only few meaningful differences were found between symptomatic and asymptomatic sides and across load conditions. This aspect thus should be further investigated in a larger patient group. However, it is of interest to note that the symptomatic side of the tested participants had a slightly lower glenohumeral translation than the asymptomatic side (Table 1). In addition, it also remains to be determined whether asymptomatic shoulders have incidental findings of rotator cuff tears, as these are common in the general population and positively associated with age, often resulting from degenerative processes [30,31]. Since almost all analysed patients reported a mild or annoying pain of the symptomatic side, a possible explanation might be that shoulder pain could lead to more controlled arm movements where activation of the uninjured rotator cuff and deltoid muscles may be more regulated, and the stabilization of the humeral head in the glenoid and/or the starting position is already more superior, allowing for less translation because of anatomic restraints by the acromion. Additionally, greater handheld weights led to greater glenohumeral translations on both the symptomatic and asymptomatic sides (Table 1). A greater load presumably leads to a greater muscle activation of the deltoid and of the supraspinatus (if the tendon is intact), with possible interference of the normal force ratio between the rotator cuff and deltoid muscles; thus, maintaining joint centring might be more difficult.

In conclusion, the presented fluoroscopy-based method for assessing glenohumeral translation during 30° abduction tests in the scapular plane has a moderate to good reliability (interrater and intrarater ICC values between 0.688 and 0.803). Moreover, this method has low radiation exposure (effective dose < 0.01 mSv), does not require additional CT scans, and is applicable in the clinic. The proposed method will allow the further investigation of shoulder joint kinematics in pathological populations.

## Figures and Tables

**Table 1:** Average translation ranges between raters 1 and 2 for the asymptomatic and symptomatic side of the ten patients for each handheld weight. (Superior—positive glenohumeral translation; inferior—negative glenohumeral translation).

Load	Abduction Translation (mm)		Adduction Translation (mm)	
	Asymptomatic Mean (SD)	Symptomatic Mean (SD)	Asymptomatic Mean (SD)	Symptomatic Mean (SD)
0 kg	3.3 (2.2)	2.3 (1.5)	–3.4 (1.9)	–1.9 (1.2)
2 kg	3.8 (2.1)	3.9 (2.6)	–3.5 (1.4)	–3.1 (1.9)
4 kg	4.1 (1.8)	3.8 (2.2)	–3.9 (1.3)	–3.4 (2.3)

SD—standard deviation.

## Data Availability

The data presented in this study are available on request from the corresponding author. The data are not publicly available due to privacy or ethical restrictions.
